# Isolation, Fermentation, and Application of Probiotics Derived from Fermented Grains

**DOI:** 10.1021/acs.jafc.5c09701

**Published:** 2026-04-20

**Authors:** Dandan Guo, Luyang Zhang, Wei Chen, Baiyan Wang, Yi Yang, Aifang Li, Shuxuan Li, Yu Huang, Yanxia Feng, Shuying Feng

**Affiliations:** † Medical College, 232830 Henan University of Chinese Medicine, Zhengzhou 450046, China; ‡ Henan Engineering Research Center for Chinese Medicine Foods for Special Medical Purpose, Zhengzhou 450046, China

**Keywords:** PDFC, fermentation, disease prevention

## Abstract

An increasing number of individuals are embracing a nutritious diet that revolves around fermented cereals, and probiotics derived from fermented cereals (PDFC) have transcended their traditional role in food fermentation, demonstrating extensive medical potential in the prevention, management, and control of various human diseases, as well as in health maintenance. The purpose of this article is to investigate the fermentation techniques of PDFC utilizing pivotal cereals such as rice, wheat, sorghum, oats, and others as raw materials. Furthermore, it aims to explore the applications of PDFC in the prevention, therapy, and health preservation of human and animal diseases, although acknowledging that the underlying mechanisms require further elucidation. Additionally, this review presents a discussion and a future outlook regarding various aspects, including the effective regulation of the fermentation process of PDFC, the maximization of their health potential, and the development of postbiotics.

## Graphical abstract

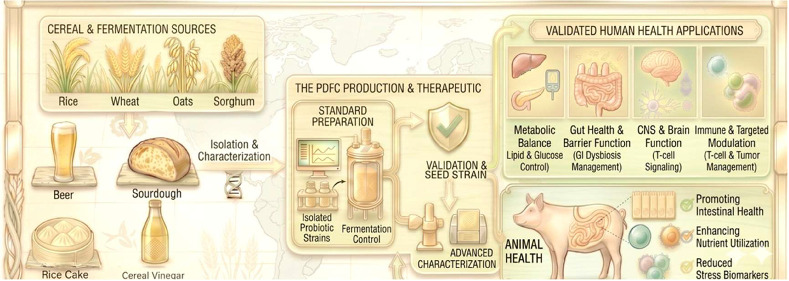

## 1 Introduction

Globally, cereals remain a primary dietary source for humanity, yet their inherent nutritional and sensory properties still warrant further improvement.^1^ Consequently, most cereals undergo fermentation prior to consumption to improve nutritional composition, extend shelf life, enhance digestibility, and optimize palatability.^2^ The isolation of probiotics from fermented cereals has recently become a significant strategy for expanding functional microbial resources and supporting the health industry.^3,4^ Here, “PDFC”, probiotics derived from fermented cereals, primarily refer to probiotic strains isolated from traditional fermented grain foods (such as Chinese sour porridge or African boza), which possess a natural adaptability to grain environments. This approach addresses the limitations of traditional dairy-derived probiotics by providing alternatives suitable for lactose-intolerant and vegetarian populations,^2^ while also identifying indigenous strains adapted to diverse cereal substrates. Research focuses on securing probiotic resources with enhanced host adaptability and distinct functional properties.^5^ Research in this area prioritizes precise matching between probiotic strains and cereal substrates, using grains as both fermentation substrates and natural prebiotics to develop effective synbiotic systems.^6^ Additionally, this strategy contributes to sustainable food systems by promoting plant-based probiotic products that reduce environmental impact and guide food biotechnology toward greater inclusivity and precision.^7^

In Africa and Asia, rice, wheat, and sorghum—serving as the primary staple crops—have become important sources for isolating PDFC, owing to their rich nutritional composition and unique fermentation characteristics. Common isolates include *Lactobacillus*, *Lactococcus*, *Bifidobacterium*, yeasts, and *Weissella*.^5^ These strains are not only employed to enhance traditional fermented foods but also demonstrate considerable potential in human disease prevention and treatment, health maintenance, and animal health promotion.^8,9^ Research into *Lactobacillus* and *Bifidobacterium* has been pervasive.^10^ In livestock farming, PDFC are also extensively employed to promote animal growth, improve gut health, and enhance immune function.^11^ Despite recent advances, significant technical challenges persist in the field.^12^ During the isolation phase, inadequate strain purity restricts the safety of subsequent applications.^3,4^ The fermentation process is frequently hindered by contamination, microbial imbalance, poor reproducibility, and low strain survival rates, all of which compromise the stability and efficacy of the resulting products.^12,13^ The mechanisms underlying the activity of certain strains during fermentation remain insufficiently understood, and some strains may adversely affect final product quality.^14^ In medical contexts, while PDFC have shown promise for treating metabolic disorders and gastrointestinal­(GI) diseases in humans, the mechanisms by which these probiotics exert their therapeutic effects in specific diseases require further investigation, and there are safety concerns regarding their use in immunocompromised individuals.^15,16^ Consequently, future research should systematically optimize the isolation, fermentation, and delivery technologies of PDFC to enhance its safety, stability, and functionality in various applications. Such advancements will support the sustainable development of the food industry, promote human health, and advance animal husbandry.

This paper reviews techniques for isolating and identifying PDFC and details fermentation processes for probiotics in key cereals, including rice, wheat, oats, and sorghum. It explores the diverse applications of PDFC in fermented foods, human and animal health care, and disease management. The review highlights challenges encountered in these application domains and proposes potential solutions, offering a valuable reference for scholars and practitioners. Ultimately, this work aims to advance our understanding of the potential of PDFC and guide future developments in the field.

## 2 Isolation and Identification of PDFC

Traditional isolation strategies employ various methods such as selective media, inhibitors, growth temperature regulation, and oxygen content adjustment to enrich and separate strains based on their cultivability.^17,18^ These approaches are well-established and remain a classic, irreplaceable approach for obtaining pure culture strains and studying their physiological characteristics. However, these processes are time-consuming and prove challenging for exploring complex, uncultivable microbial communities.^19^ Nonculture-dependent techniques have overcome this bottleneck by circumventing the cultivation step, enabling direct in situ identification and physical sorting of target cells within complex samples, such as fermented cereal matrices.^20,21^ Among these, flow cytometry-based fluorescence in situ hybridization employs specific fluorescent probes targeting 16S rRNA to achieve targeted sorting of strains with known genetic backgrounds.^20^ In contrast, single-cell Raman spectroscopy analyzes the intrinsic “chemical fingerprint” of cells, enabling the selection of viable bacteria based on metabolic phenotypes (such as activity and function) without prior labeling.^21^ These techniques enable the direct and efficient identification of probiotics with specific functionalities or genetic characteristics from fermented cereals.

The identification and characterization of bacterial strains combines traditional and modern molecular biology techniques, forming a systematic analytical workflow.^22^ At the phenotypic level, preliminary differentiation is first performed by observation of colonies and cell morphology (e.g., Gram staining),^22^ followed by physiological and biochemical experiments (e.g., catalase reaction, sugar fermentation profiling, and salt tolerance testing) to further elucidate their metabolic properties.^23^ Among these, the catalase test is primarily employed for preliminary screening of specific bacterial groups, particularly distinguishing lactic acid bacteria­(LAB) (which yield negative results) from non-LAB.^23^ In contrast, modern identification relies on molecular biology techniques. The 16S rRNA gene sequence analysis is considered to be the gold standard for bacterial identification, enabling precise classification at the genus or species level. However, this method has limited resolution for distinguishing closely related strains.^4^ For fungal identification, including yeasts, ITS sequence analysis and 18S rDNA and 28S rRNA sequencing are commonly employed.^24,25^ Whole-genome sequencing, also regarded as the gold standard, is applicable to both bacteria and fungi.^4^ It enables precise strain-level identification, tracing evolutionary relationships through single-nucleotide polymorphism analysis, and comprehensive examination of functional genes.^4^ Additionally, matrix-assisted laser desorption/ionization time-of-flight mass spectrometry­(MALDI-TOF MS) is widely used in routine laboratory identification due to its rapid and high-throughput protein fingerprinting capabilities.^3^ The accuracy of MALDI-TOF MS depends on the quality and completeness of the reference database, which may limit its ability to identify newly discovered or rare strains.^3^ These methods are complementary and, when used together, establish a comprehensive system for strain identification and characterization from phenotype to genotype, providing technical support for the in-depth exploration of probiotic resources in fermented cereals.

## 3 Fermentation Technology of PDFC

In most countries across Asia, Africa, and Europe, rice, wheat, oats, and sorghum are regarded as key cereal crops.^26–29^ The nutrient-rich nature of grains not only promotes a wide range of beneficial physiological effects but also stimulates the growth of indigenous probiotics, such as LAB and *Bifidobacterium*, which are natural cultures.^30^ PDFC enhance the flavor and nutrient content of fermented grains, as well as boost the immune system and intestinal health of the local populations.^31^ Compared with conventional probiotics, PDFC offer numerous advantages.^32,33,38,40,41^ First, many PDFC originate from traditional fermented grain foods with a long history of consumption, providing solid “history of use” evidence for their safety assessment.^32^ Second, the ecological stresses of the grain matrix have shaped the strains’ stronger stress resistance.^33^ Compared to other growth media, cereal-based environments typically have lower pH buffering capacity, a complex carbohydrate composition, and potential antimicrobial substances (such as polyphenols).^34–37^ Strains that have adapted to such environments over the long-term exhibit superior tolerance.^33^ For example, *Saccharomyces cerevisiae* ( *S. cerevisiae* ) and *Kluyveromyces marxianus* isolated from the traditional African cereal food mawè maintained slow metabolic activity even under extreme acid stress at pH 2.5, whereas the growth of the same yeast species from other sources was largely inhibited.^33^ This indicates that within the same species, differences in isolation source can lead to significant divergence in key tolerance traits, and PDFC generally retain a complete spectrum of probiotic functions.^33^ Furthermore, PDFC have adapted to the grain environment over time and evolved a unique system of carbohydrate-active enzymes.^38^ Compared with conventional probiotics, they are more efficient at degrading key components specific to grains (such as arabinan and β-glucan) and produce a unique profile of metabolites characteristic of grains.^27,38^ —a capability that exhibits significant strain specificity.^39^ For example, LAB isolated from wholegrain foods release ferulic acid—a compound with antioxidant activity—from the grain matrix via feruloyl esterase­(FAE).^40^ Further studies have shown that probiotic fermentation of wheat bran can convert ferulic acid into more bioactive derivatives.^41^ This ability to “solubilize” and “metabolize” cereal substrates is a key characteristic that distinguishes PDFC from strains sourced from other origins.^40–43^ Table 1 outlines the functional mechanisms of PDFC and their applications in disease intervention. However, regional variations in environmental conditions, different cereal substrates (prebiotics), and fermentation methods all influence the types of probiotics present and the efficacy of fermentation during the process.^6^ Consequently, there is an urgent need to consolidate probiotic fermentation technologies for various cereals (see Figure 1), compile relevant experience, and document key information. The following sections briefly review probiotic fermentation technologies for rice, wheat, sorghum, and oat fermentation products.

**Figure 1:**
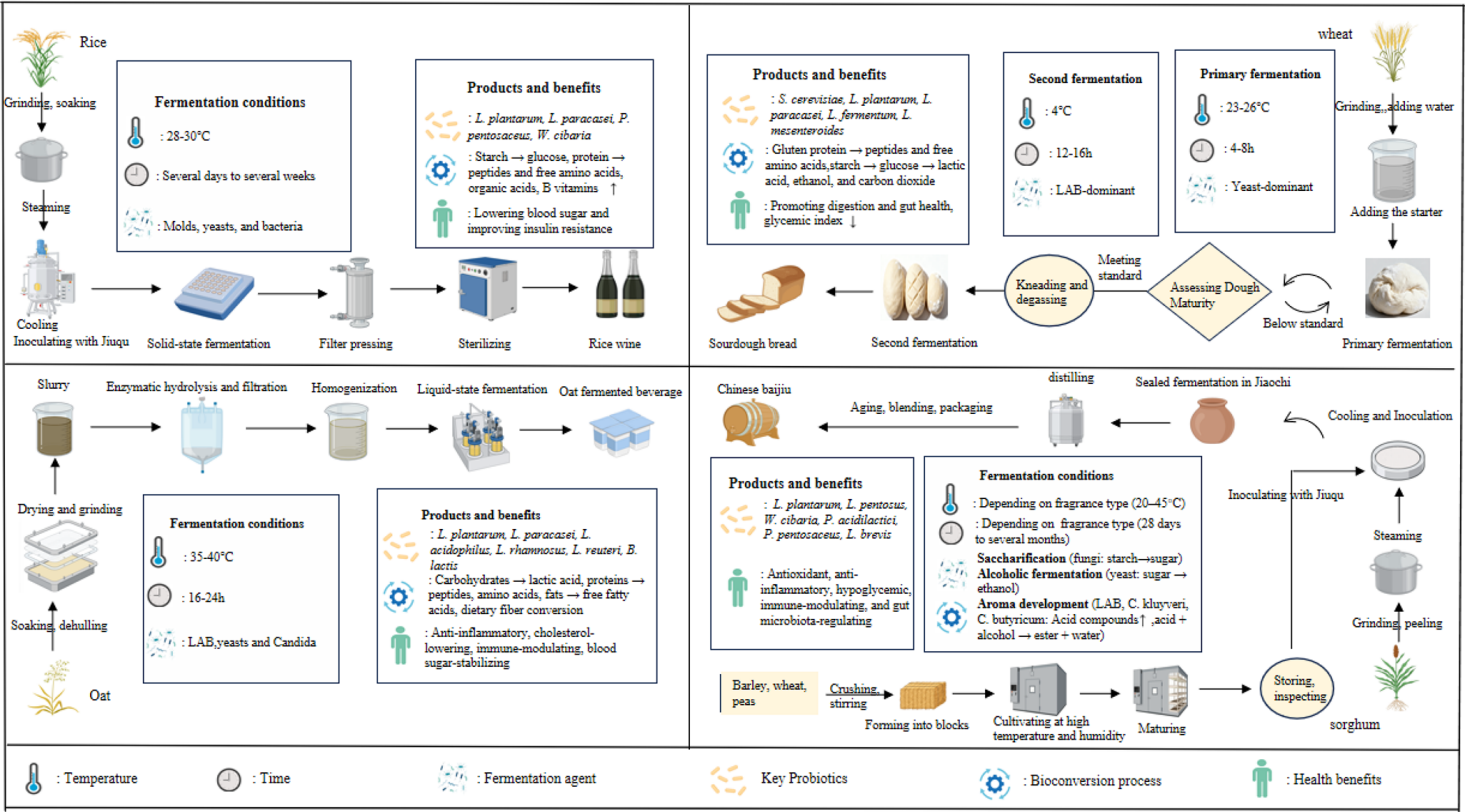
Four PDFC fermentation technologies. (a) Rice probiotic fermentation employs solid-state/semisolid-state processes. The core procedure involves soaking and steaming rice, inoculating with *A. oryzae* for saccharification, and yeast fermentation and pressing. Precise temperature control ensures thorough saccharification, producing a low-alcohol, sweet-and-sour balanced probiotic beverage. (b) Wheat probiotic fermentation centers on solid-state fermentation using yeast (e.g., *S. cerevisiae*). The gases produced cause dough to rise and promote the gluten network formation. The process flow includes mixing, proofing, and baking. Precise control of temperature and time (avoiding under-/overfermentation) ensures a light, airy texture with desirable fullness. (c) Oat probiotic fermentation primarily employs liquid fermentation. Raw materials are processed via enzymatic hydrolysis or steaming and then inoculated with LAB and yeast for synergistic lactic and alcoholic fermentation. The final product is obtained through blending. Key controls include preventing contamination and precisely determining the fermentation end point to preserve oats’ nutrient-rich properties, such as high β-glucan content. (d) Sorghum probiotic fermentation (exemplified by Chinese baijiu) employs a unique combination of solid-state and liquid fermentation. A complex microbial consortium (molds, yeasts, and bacteria) performs synergistic saccharification and alcoholic fermentation, yielding rich flavor compounds. The process flow includes grinding, steaming, cooling, starter addition, pit fermentation, and distillation. Meticulous pit management (temperature and humidity) and proper distillation produce a high-alcohol, full-bodied spirit.

**Table 1:** Functional Mechanisms of PDFC and Their Applications in Disease Intervention^a^

cereal	cereal fermented products	PDFC	mechanisms of action of probiotic	human diseases	references
rice	fermented rice water	*Leuconostoc* and *Weissella*	upregulating genes related to nutrient absorption (MCT-1 and MCT-2) and barrier integrity (occludin and ZO-1)	NA	^6^
rice	fermented rice beer	*Bacillus* sp. FRB_A and *Acetobacter* sp. FRB_B	possessing antioxidant and anti-α-glucosidase activity	diabetes	^43^
rice	indigenous ethnic fermented products	*Pediococcus pentosaceus, Limosilactobacillus fermentum,* and *Lactiplantibacillus plantarum*	competing with pathogens for nutrients while generating antimicrobial compounds	NA	^44^
rice	rice wine	*Lactiplantibacillus plantarum, Pediococcus pentosaceus,* and *Weissella cibaria*	showing potent α-glucosidase inhibition	diabetes	^45^
rice	atingba	*Lactiplantibacillus plantarum*	producing antimicrobial metabolites and inhibiting the formation of pathogenic biofilms	NA	^46^
rice	Hongqu rice wine	*Limosilactobacillus fermentum* FZU501	inhibiting CYP2E1 and promoting cholesterol excretion	alcoholic liver injury	^47^
rice	fermented rice	*Bifidobacterium* sp. MKK4	promoting lipolysis and inhibiting lipogenesis	obesity	^48^
rice	fermented rice	*Lactobacillus*	accelerating fatty acid breakdown (upregulating ACO/CPT1) and inhibiting fat synthesis (downregulating FAS)	obesity, depression	^49^
rice	torani	*Lactobacillus*	regulating gut microbiota	*Clostridium difficile* infections	^50^
rice	rice-based beverages	*Limosilactobacillus fermentum* MG7011	antioxidant, anti-inflammatory, and gut barrier enhancement	NA	^51^
brown rice	fermented grains	*Lacticaseibacillus acidophilus* and *Bifidobacterium animalis subsp. lactis*	upregulating the expression levels of p53 and Bax in colonic tissue, and increasing the Bax/Bcl-2 ratio	colorectal cancer	^52^
wheat	traditional fermented wheat	*Lactiplantibacillus plantarum* and *Weissella confusa*	demonstrating antimicrobial secretion, comprehensive antioxidant activity, and glutathione enhancement	NA	^53^
wheat	traditional fermented dough	*Saccharomyces cerevisiae, Torulaspora delbrueckii, Kluyveromyces marxianus,* and *Saccharomyces boulardii*	producing antimicrobial compounds, synthesizing folate, and absorbing cholesterol	hypercholesterolemia	^54^
sorghum	ogi-baba and pito	*Pediococcus pentosaceus* and *Lactiplantibacillus plantarum*	producing antimicrobial metabolites lactic acid, diketone and hydrogen peroxide, and enhancing β-galactosidase activity	lactose intolerance	^55^
sorghum	ting	*Lacticaseibacillus helveticus, Lacticaseibacillus amylolyticus, Lacticaseibacillus paracasei, Lactiplantibacillus plantarum, Levilactobacillus brevis,* and *Loigolactobacillus coryniformis*	suppressing intestinal pathogens via competitive exclusion and antimicrobial secretion	NA	^56^
sorghum	sorghum-based traditional fermented food	*Lactiplantibacillus plantarum* and *Lactiplantibacillus pentosus*	producing bacteriocins and assimilating cholesterol	NA	^57^
red rice and white rice	traditional fermented sour rice water	*Pediococcus pentosaecus* and *Lactococcus lactis*	releasing diverse antimicrobial substances	NA	^58^
barley, corn, millet, and oats	boza	*Limosilactobacillus fermentum* and *Levilactobacillus brevis*	exerting antioxidant effects through DPPH radical scavenging	NA	^59^
sorghum, wheat, and rice	soy sauce aroma type baijiu	*Lactiplantibacillus pentosus* LTJ12, *Pediococcus acidilactici* LTJ28, *Lactiplantibacillus plantarum* LTJ30, and *Pediococcus acidilactici* LTJ32	promoting alcohol metabolism and repair the gut-liver axis	alcoholic liver injury	^60,61^
sorghum, wheat, and rice	soy sauce aroma type baijiu	*Lactiplantibacillus pentosus*	modulating inflammation and immunity and promoting alcohol metabolism	NA	^62^
sorghum, corn, rice, wheat, and millet	traditional Chinese baijiu	*Weissella cibaria* (OP288150), *Pediococcus acidilactici* (OP288151), *Pediococcus pentosaceus* (OP288154), *Pediococcus pentosaceus* (OP288156), and *Levilactobacillus brevis* (OP288158)	possessing α-amylase and α-glucosidase inhibitory activity	diabetes	^63,64^
wheat and rye	sourdough	*Kluyveromyces marxianus*	alleviating cellular damage induced by Candida albicans and suppressing the expression of the SAP2 and SAP6 genes	vulvovaginal candidiasis	^65^
rice and buckwheat	fermented rice buckwheat	*Bacillus* DU-106, Lactiplantibacillus plantarum	inhibition of hepatic cholesterol synthesis and lipid production	hyperlipidemia	^66^
millet and sorghum	fermented grain beverage	NA	generating ACE-I peptides	hypertension	^67^
black glutinous rice, fragrant rice, rice, broomcorn millet, and millet	fermented grain beverage	LAB and yeast	reducing oxidative stress	intestinal mucositis	^68^
black barley and quinoa	fermented grains	LAB	anti-inflammatory and enhancing blood-brain barrier and colonic barrier	neurodegenerative diseases	^69^

a NA, Not available; CYP2E1, cytochrome P450 family 2 subfamily E member 1; ACO, acyl-CoA oxidase; CPT1, carnitine palmitoyltransferase 1; FAS, fatty acid synthase; Bcl-2, B-cell lymphoma 2; Bax, Bcl-2-associated X protein; Caspase-3, cysteine-aspartic protease 3; DPPH, 2,2-diphenyl-1-picrylhydrazyl; and ACE-I peptide, angiotensin-converting enzyme inhibitory peptide.

### 3.1 Rice Probiotic Fermentation

Rice fermentation relies primarily on lactic acid and alcoholic fermentation, and the choice of process directly determines end-product characteristics (e.g., acidity and flavor) and probiotic functionality.^31^ Based on the form of the substrate, fermentation can be classified into two categories: liquid fermentation and solid-state fermentation.^70,71^ Liquid fermentation is exemplified by rice wine, rice vinegar, and fermented rice beverages.^71–73^ Such processes are usually carried out under partially anaerobic or anaerobic conditions using a liquid or semisolid substrate, and are centered on the synergistic or sequential action of yeasts and LAB (e.g., *Lactobacillus*).^62^ The typical production method for Korean makgeolli involves cooling steamed rice to 30–35 °C, inoculating it with saccharification agents such as molds and LAB to initiate simultaneous saccharification and fermentation, and maintaining the mixture in sealed containers for approximately 48–72 h.^74^ The critical control points in this process include the initial sugar content, the inoculation sequence, and the fermentation temperature.^74^ In contrast, solid fermentation processes (e.g., fermented rice noodles) predominantly rely on complex microbial communities naturally inoculated under open, ambient-temperature conditions.^71^ Although this method faces challenges regarding batch-to-batch consistency, the intricate microbial interactions generate a richer array of flavor compounds and bioactive components (e.g., γ-oryzanol and polyphenols),^75^ which synergistically enhance functional properties like antioxidant property.^75,76^

Despite the different pathways, both fermentation techniques systematically hydrolyze the starch and small amounts of protein in rice through microbial-secreted amylase and protease, yielding oligosaccharides, monosaccharides, amino acids, and small-molecule peptides.^14^ This process lowers the glycemic index, enhances the bioavailability of nutrients, and generates organic acids such as lactic acid and acetic acid.^58,77^ These compounds confer a distinctive tangy flavor while effectively inhibiting pathogenic bacterial growth.^77^ The probiotic potential of these fermented rice products has been preliminarily validated.^77^ For instance, *Lacticaseibacillus lactis* and *Lactiplantibacillus pentosus* (*L. pentosus*) isolated from fermented sour rice water have demonstrated excellent acid and bile salt tolerance, along with strong intestinal adhesion capacity in vitro.^58^ Traditional production methods continue to depend on unstable natural fermentation.^14^ The specific functional microbial strains involved remain unidentified, which increases the risk of microbial contamination and the accumulation of mycotoxins.^78,79^ Studies indicate that *Burkholderia gladioli pathovar cocovenenans* in wet rice noodles from the Guangzhou region proliferates more rapidly at elevated temperatures of 26 and 32 °C, leading to increased bacillomycin accumulation.^79^ Additional analyses have shown that rice and rice products contain varying levels of total mercury and methylmercury; however, current concentrations do not present a health risk.^78^ Future technological advancements must be grounded in an understanding of fermentation mechanisms,^72–75^ utilizing fermenters containing functional strains to specifically enhance the efficacy of probiotics,^58^ while simultaneously strengthening prevention and control measures across the entire supply chain to ensure product safety and consistent quality.^58^ Additionally, research should focus on strains that efficiently utilize processing byproducts, such as rice bran,^79^ to achieve comprehensive valorization.

### 3.2 Wheat Probiotic Fermentation

Wheat, rich in gluten protein and starch, is mainly used in both sourdough fermentation and grain beverage brewing, requiring differentiated microbiological strategies for the production of functional products such as breads, beers, and wines.^80–82^ Sourdough exemplifies solid-state fermentation of wheat and serves as a microecosystem in which LAB and yeast coexist symbiotically.^83^ Fermentation occurs through either natural inoculation or the use of sourdough starter, typically at room temperature under microaerophilic conditions for durations ranging from several hours to several days.^83^ LAB metabolize organic acids, reducing the dough’s pH to below 5.0. This acidification inhibits unwanted bacteria and activates endogenous proteases that degrade gluten proteins into more readily absorbed amino acids and peptides, thereby enhancing digestive tolerance.^83,84^ Concurrently, carbon dioxide produced by yeast contributes to the dough’s characteristic fluffy texture.^84^ Although sourdough retains grain nutrients intact, its dependence on unstable microbial communities, resulting in poor batch consistency,^85^ and the risk of contamination with mycotoxins (e.g., deoxynivalenol and zearalenone) in the raw materials that can be present throughout the entire industrial chain,^13^ are key bottlenecks constraining its safety.

Liquid fermentation of wheat, as seen in beer and Huangjiu, focuses on generating flavor compounds and ethanol.^81,82^ Huangjiu brewing utilizes a dual-stage process that integrates solid-state koji production with subsequent liquid fermentation.^81^ Malted barley, which contains *Mucor*, *Aspergillus, Bacillus*, and LAB, provides saccharification enzymes and undergoes primary fermentation with yeast at 30 °C, followed by secondary maturation at 15 °C.^86^ This process extends over several months.^86^ In contrast, beer brewing employs pure yeast strains that ferment under more controlled temperature conditions.^87^ Wheat malt amylases and proteases convert starch and proteins into sugars and amino acids, which serve as substrates for yeast fermentation and as precursors for the Maillard reaction, resulting in the formation of various aromatic compounds.^88^ Trace organic acids produced by LAB contribute significantly to the overall flavor profile.^88^

Traditional Huangjiu production is characterized by complex processes and extended fermentation cycles, while industrial beer production emphasizes efficiency, often resulting in reduced flavor complexity.^88^ Current technological advancements focus on developing synergistic fermentation strategies that combine non-*Saccharomyces* with LAB to enhance flavor complexity in a targeted manner.^89,90^ In parallel, unpasteurized craft beers are being explored as potential vehicles for delivering functional probiotic bacteria; however, ensuring food safety remains a critical prerequisite for their industrial-scale production.^91,92^ The diversity of wheat fermentation technologies demonstrates the varied utilization of a common substrate by different microorganisms.^80–82^ Future research should prioritize the development of defined synthetic microbial communities with defined functions and the implementation of a comprehensive mycotoxin monitoring system spanning from raw materials to finished products.^14^ The integration of multiomics technology is essential for achieving synergistic improvements in the flavor, nutritional value, and safety of wheat fermentation products.

### 3.3.Oat Probiotic Fermentation

Oat probiotic fermentation technology aims to leverage the substrate properties of its high β-glucan and polyphenol content.^93^ Through specific strain selection and process control, high-cell-density probiotic cultivation is achieved, along with the production of functional metabolites.^93,94^ The effectiveness of this approach depends on the precise regulation of interactions among microbial strains, fermentation processes, and substrates.^94^ In LAB-dominant fermentation, strains such as *Lactiplantibacillus plantarum* (*Lp. plantarum*) and *Lactobacillus acidophilus* (*L. acidophilus*) exhibit high adaptability to oat substrates.^94,95^ In contrast, multistrain synergistic fermentation, including cocultivation of LAB with yeast or filamentous fungi, results in more complex metabolic interaction networks.^96,97^ In these systems, yeasts, particularly *Yarrowia lipolyticus* with lipolytic activity, utilize carbon sources that are not fully metabolized by LAB and secrete proteases and lipases that convert macromolecular nutrients into flavor precursors.^93^ This process enhances the intensity and harmony of the aroma profile.^93–98^ Certain filamentous fungi, such as *Aspergillus oryzae* (*A. oryzae*), have demonstrated the ability to improve dietary fiber solubility and protein digestibility through xylanase and esterase synthesis.^99^ The synergistic metabolic effects, such as yeast supplying essential growth factors for LAB, are critical for achieving nutritional fortification.^94^

Critical control points in the fermentation process include pretreatment, temperature regulation, and precise determination of the fermentation end point.^100–102^ Common pretreatment methods for oat fermentation include enzymatic hydrolysis, germination, grinding, and drying.^103^ The substrate undergoes sterilization and is subsequently stored at 4 °C for approximately 20 days.^103^ The fermentation temperature is typically maintained at 30–40 °C.^93,102,104^ The live bacterial count and acid production reach their peak after 16–18 h. Prompt termination of fermentation at this stage, followed by rapid cooling, is essential for producing highly active products. Subsequent storage at 4 °C preserves microbial viability.^92^ Despite advancements, this technology continues to encounter several challenges.^104,105^ The principal concern is the survival rate of live bacteria; energy depletion required to maintain intracellular pH during storage is the primary mechanism responsible for the rapid decline in viable bacterial counts and compromised storage stability.^104^ The addition of specific sweeteners that do not interfere with fermentation, glucose to enhance bacterial proliferation and lactic acid production, honey, and milk to support probiotic growth activity has proven effective in addressing these issues.^102,103^ Nevertheless, oat-based probiotic beverages have a shorter shelf life and lower sensory acceptability than dairy products.^105^ Research to date has predominantly concentrated on LAB, with limited investigation into yeast and other microbial strains.^94^ Future technological advancements are expected to concentrate on two primary areas.^93,94^ The first area involves establishing a dedicated strain library by selecting specialized probiotics from traditional fermented products to achieve efficient growth and enhanced sensory qualities in oat-based matrices.^93^ The second area focuses on process innovation, including the exploration of fermentation strategies based on feedback control systems such as pH-stat and the development of microencapsulation techniques to address challenges related to bacterial stability.^94^ The overarching objective is to develop a standardized technical solution that consistently produces high-activity and high-efficacy oat probiotic products without the need for added sweeteners.

### 3.4 Sorghum Probiotic Fermentation

Sorghum probiotic fermentation technology employs targeted microbial strategies to address matrix limitations caused by antinutritional factors such as tannins.^106^ This approach converts the substrate into functional foods enriched with probiotics and functional metabolites, including short-chain fatty acids­(SCFA) and gamma-aminobutyric acid­(GABA).^98^ Technical pathways are generally classified as either solid-state sourdough fermentation or compound fermentation, depending on the intended end product.^107,108^ Solid-state sourdough fermentation, primarily mediated by LAB, is used to produce staple foods such as Ingera.^109^ This process generally involves controlled fermentation at ambient temperatures, emphasizing the inoculation or enrichment of specific LAB.^97^ Through homofermentative and heterofermentative pathways, LAB produce organic acids that lower the system’s pH.^107^ The resulting acidic environment inhibits contaminant and opportunistic microorganisms and activates endogenous phytase and tannic acid hydrolase, significantly reducing the phytate and tannin content. Consequently, there is a significant reduction in phytate and tannin content, resulting in fermented sorghum flour with substantially lower phytate and oxalate levels compared to unprocessed samples.^107,110^ Simultaneously, acidic conditions activate proteases and esterases, facilitating the degradation of macromolecules such as starch and proteins.^63^ These biochemical processes enhance nutrient bioavailability and improve the texture of sorghum.^110,111^ Furthermore, the exopolysaccharides­(EPS) produced by LAB and other microbes, in conjunction with changes in protein structure, enhance dough water retention capacity and significantly improve its rheological properties (viscoelasticity) and sensory qualities.^107^

Compound fermentation systems employ multiple microbial communities that interact synergistically to achieve efficient simultaneous saccharification and the generation of complex flavor compounds.^58,59^ This methodology is applied in the production of beverages such as sorghum liquor, where the primary technology involves a solid-state process followed by liquid-state compound fermentation.^108^ A notable example is the brewing technique of Fengxiang liquor.^108^ The process initially cultivates a complex microbial community, including filamentous fungi (*Aspergillus*), yeast, and bacteria, through open solid-state fermentation using medium-temperature Daqu.^108^ The resulting Daqu is then combined with sorghum and subjected to several months of liquid-dominant fermentation in sealed fermentation pits.^108^ In this system, amylase and saccharifying enzymes produced by filamentous fungi are essential for initiating fermentation.^112^ These enzymes systematically saccharify sorghum starch, generating substrates for yeast and LAB that cannot directly utilize starch.^112,113^ Yeast subsequently dominates ethanol fermentation,^112^ while LAB and acetic acid bacteria are responsible for lactic acid and acetic acid fermentation, respectively.^113^ This sequential division of labor and synergy among microbial communities establishes the complex flavor profile, including alcohols, acids, and esters, in the beverage.^112,113^

Sorghum fermentation encompasses diverse methods, yet its primary aim is to improve edibility and functionality through microbial processes.^114^ The efficacy of probiotic strains derived from various sorghum fermentation products has also been demonstrated.^64,65^ Despite these advances, the system faces notable constraints.^106^ Dependence on natural fermentation leads to unpredictable microbial populations, which can compromise product safety and consistency.^106,109^ Additionally, limited strain availability restricts the effective use of yeast and fungi for fiber degradation and flavor compound synthesis, thereby constraining both flavor complexity and opportunities for nutritional improvement.^106,113^ Future technological advancement requires a transition from empirical control to precision design.^112,113^ Key strategies include targeted selection and breeding of PDFC with defined functions, such as efficient degradation of antinutritional factors, synthesis of EPS, and production of flavor compounds.^113^ Additionally, due to sorghum’s nutritional deficiencies in provitamin A compounds such as β-carotene, diversified matrix blending based on sorghum should be pursued to optimize both nutrition and flavor.^106^

## 4 Application of PDFC and Their Metabolites in Human Diseases

Common PDFC encompass *Lactobacillus, Lactococcus, Bifidobacterium, Bacillus, Saccharomyces,* and *Weissella.* ^5^ Their probiotic functions depend not only on direct regulation of host pathways by the bacterial cells themselves,^48^ but also closely relate to their abundant metabolic products. In addition to conventional metabolites such as lactic acid, SCFA, and GABA,^115–118^ PDFC can also produce a class of specific functional molecules closely associated with the cereal matrix, such as angiotensin-converting enzyme inhibitory­(ACE-I) peptides released through the hydrolysis of cereal proteins, and α-glucosidase inhibitors synthesized during fermentation.^45,64,67^ Simultaneously, enzymes secreted by PDFC (such as glycosidases, esterases, and phenolic decarboxylases) biotransform grain components, releasing and converting bound polyphenols into phenolic derivatives with anti-inflammatory, antioxidant, and anticancer activities.^116,119^ These strains can form a “lock-and-key” synbiotic system with structurally specific prebiotics found in grains, working synergistically to optimize the gut microbiota and produce a biological effect where “1 + 1 > 2.” ^38,121,122^ It is worth noting that this metabolic capacity varies significantly among different strains, highlighting the importance of screening for functional strains.^121,123,124^ Consequently, PDFC demonstrates unique systemic and applied potential in intervening against diverse diseases, including metabolic disorders, GI diseases, central nervous system­(CNS) diseases, and cancer. The mechanism by which PDFC confer health benefits is illustrated in Figure 2.

**Figure 2:**
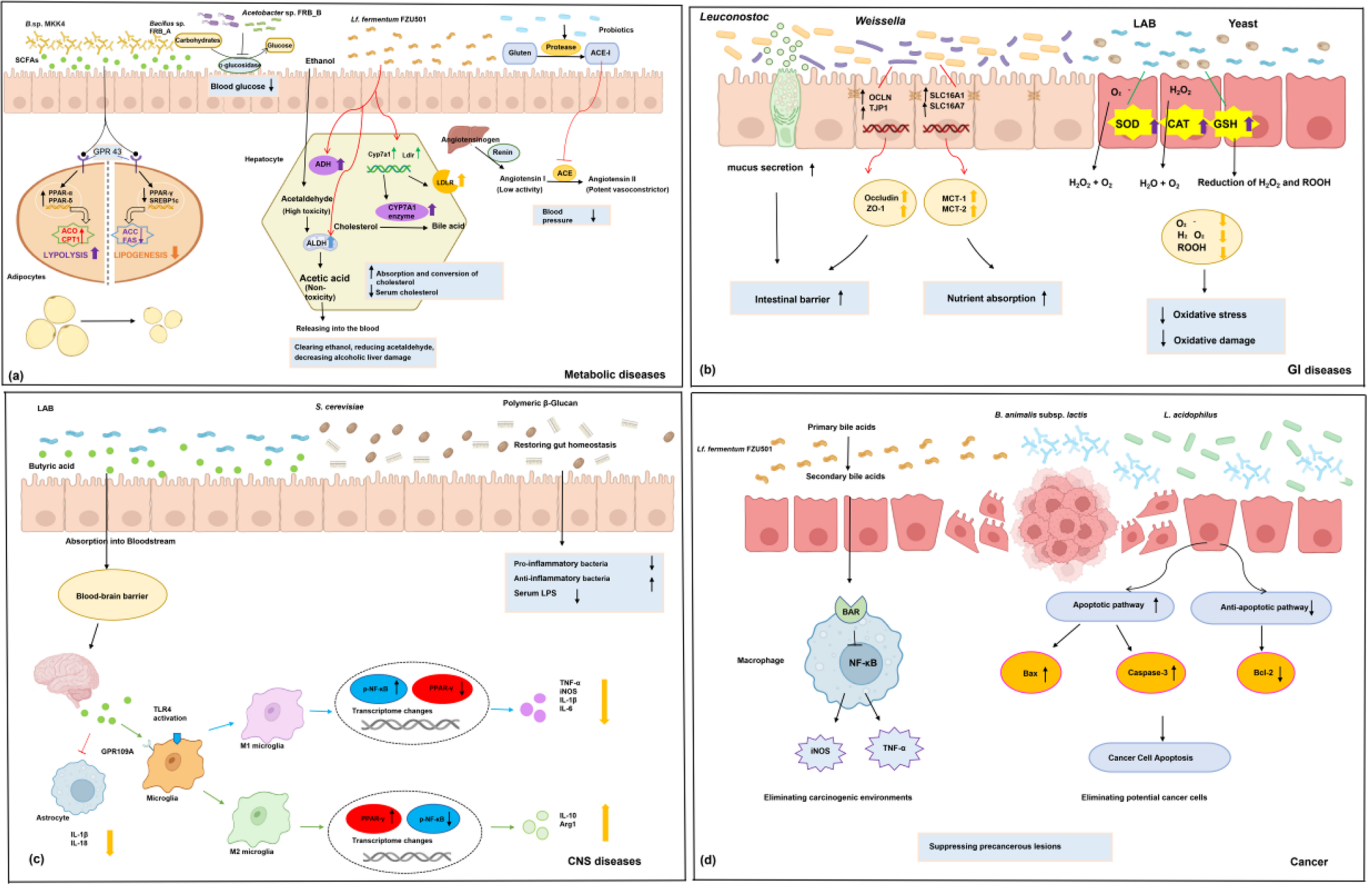
PDFC confer health benefits through multifaceted mechanisms. (a) *B*.sp. MKK4 fermented rice produces SCFAs that activate the GPR43 receptor in adipocytes, upregulating the expression of lipolysis-related transcription factors PPAR-α and PPARδ while suppressing the expression of the lipid synthesis pathway regulators SREBP-1c and PPAR-γ. This promotes lipolysis, inhibits lipogenesis, and alleviates obesity. *Bacillus* sp. FRB_A and *Acetobacter* sp. FRB_B reduces postprandial blood glucose by inhibiting α-glucosidase. *Lf. fermentum* FZU501 enhances ADH and ALDH activity, thereby promoting ethanol metabolism in hepatocytes and reducing alcoholic liver injury. *Lf. fermentum* FZU501 also promotes the expression of the Cyp7a1 and Ldlr genes in hepatocytes, thereby enhancing cholesterol uptake and conversion. Certain probiotics can degrade cereal proteins into ACE-I peptides, inhibiting the conversion of angiotensin I to angiotensin II. This reduces vasoconstrictive substances, thereby alleviating hypertension. (b) *Leuconostoc* and *Weissella* promote the expression of tight junction protein genes (OCLN and TJP1) and nutrient absorption genes (SLC16A1 and SLC16A7) in intestinal epithelial cells. LAB and yeast enhance the expression of antioxidant enzymes (SOD, CAT, and GSH) in intestinal cells, thereby reducing excessive reactive oxygen species, alleviating oxidative stress, and mitigating intestinal inflammation. (c) Butyric acid, produced by LAB fermentation of black barley and quinoa, is absorbed into the bloodstream through the gut. It crosses the blood-brain barrier to inhibit excessive astrocyte activation and promote a shift in microglia from proinflammatory M1 to anti-inflammatory M2 phenotypes, thereby reducing neuroinflammation. High-molecular-weight β-glucan produced by *S. cerevisiae* reduces serum LPS levels and decreases neuroinflammation by increasing anti-inflammatory gut bacteria and decreasing proinflammatory gut bacteria. (d) *Lf. fermentum* FZU501 promotes the production of secondary bile acids, thereby inhibiting the release of reactive oxygen species and inflammatory cytokines by macrophages, improving the procarcinogenic environment. *Bifidobacterium animalis subsp. Lactis* (*B. animalis subsp. Lactis*) and *L. acidophilus* enhance the expression of apoptosis-related proteins (upregulating Bax/caspase-3) and suppress the activation of antiapoptotic pathways, thereby eliminating potential cancer cell.

### 4.1 Metabolic Diseases

PDFC play a significant role in the prevention and treatment of metabolic diseases such as obesity, diabetes, and hypertension by regulating gut microbiota composition, microbial metabolites, and barrier function.^48,116,120,125,126^ For instance, the *Bifidobacterium* sp. (*B*. sp.) MKK4 derived from fermented rice significantly improves metabolic disorders in high-fat diet-induced obese mice.^48^ Its mechanism involves upregulating the expression of lipolysis-related transcription factors PPARα and PPARδ, while suppressing the transcriptional activity of SREBP-1c—a key regulator of the lipid synthesis pathway—and its downstream genes (acetyl-CoA carboxylase and fatty acid synthase).^48^ Notably, the cofermentation system of *B*. sp. MKK4 with rice matrix demonstrated superior efficacy in weight reduction, improvement of hepatic steatosis, and lipid regulation compared to single-strain fermentation.^48^ This enhanced effect is presumed to result from the molecular affinity of *B*. sp. MKK4 for specific components in the rice matrix, such as resistant starch with a specific structure and bound phenolics.^48^ Moreover, *Bacillus* DU-106 and *Lp. plantarum* are capable of efficiently utilizing structurally distinct dietary fibers in grains to produce a spectrum of SCFA dominated by propionic and butyric acids.^66^ These SCFA participate in central energy regulation by activating G-protein-coupled receptor 43­(GPR43) and GPR41 receptor and inhibiting neuropeptide Y-positive neurons in the hypothalamus.^115,127^ Additional research indicates that *Lactobacillus* strains derived from fermented rice not only promote fatty acid oxidation and suppress gene expression related to fat production, but also improve glucose tolerance, insulin sensitivity, and GLUT4 expression, thereby synergistically exerting antiobesity and antihyperglycemic effects.^49^ Collectively, these findings demonstrate that the functional systems formed by PDFC during cofermentation with the grain matrix hold great potential for nutritional interventions in metabolic diseases.^48,49,66^

PDFC exhibit diverse mechanisms of action in diabetes management.^45,64,128^ Beyond improving glucose metabolism by regulating the gut microbiota,^125^ another key pathway involves inhibiting the activity of intestinal carbohydrate digestive enzymes such as α-amylase and α-glucosidase.^45,64,129^ For instance, *Weissella cibaria* (*W. cibaria*) JR01, isolated from fermented foods in Guizhou, exhibited an α-glucosidase inhibition rate of 33.8% in its cell-free fermentation supernatant.^45^ Additional studies indicate that both cell-free supernatants and intracellular extracts from LAB derived from traditional Chinese liquor and fermented grains significantly inhibit these two enzymes.^64^ Notably, *Lp. plantarum* supernatant exhibited an α-amylase inhibition rate as high as 86.15%, suggesting the production of potent enzyme-inhibiting substances during fermentation.^64^ These findings suggest that the hypoglycemic effects of PDFC do not entirely depend on the intestinal colonization of live bacteria.^64^ Active components synthesized or accumulated within the bacteria during fermentation—such as acarbose-like metabolites—may play a crucial role.^64^ This mechanism provides a basis for developing probiotic-based postbiotic formulations for diabetes intervention.^63,129^ Additionally, certain strains exhibit multitarget regulatory characteristics.^43,116^ *Bacillus* sp. FRB_A and *Acetobacter* sp. FRB_B, isolated from a traditional fermented rice beer of Assam, India, not only inhibit α-glucosidase but also enhance insulin receptor sensitivity, thereby synergistically improving the symptoms of diabetic rats.^43^ Taking the finger millet fermentation system as an example, after fermentation by *Weissella confusa* 2LABPT05, α-glucosidase inhibitory activity increased by 7%, total phenolic content rose by 34.8%, and GABA content tripled, while simultaneously promoting the proliferation of *Bifidobacterium* and the production of SCFA.^128^ This multifaceted synergy stems from the strains’ comprehensive metabolic capabilities regarding bound phenolics, GABA precursors, and specific carbohydrate structures in the grain.^128^ *Lactobacillus fermentum* (*Lf. fermentum*) MF423, derived from fermented rice bran, has demonstrated enhanced antidiabetic effects in both in vitro and in vivo studies.^130^ These strains increase glucose uptake in insulin-resistant HepG2 cells and reduce lipid accumulation in these cells.^130^ Currently, the efficacy of PDFC in managing diabetes requires further evidence to support its benefits, and its effects demand further research to clarify the following: (1) screening strains capable of efficiently inhibiting α-amylase and α-glucosidase, while identifying optimal grain substrates and fermentation processes; (2) exploring synergistic combinations of probiotic strains; and (3) assessing the impact of individual gut microbiota variations on intervention outcomes.

PDFC, particularly strains originating from fermented grain alcoholic beverages, demonstrate significant potential in the prevention and treatment of alcoholic liver disease.^47,60,61^ *Lf. fermentum* FZU501, isolated from red yeast rice wine, exhibits a triple-action systemic protective effect: detoxification, fat reduction, and antioxidant activity.^47^ Regarding detoxification, it inhibits cytochrome P450 2E1, a key enzyme in the toxic alcohol oxidation pathway, while enhancing alcohol dehydrogenase 2 (ADH2) and acetaldehyde dehydrogenase 2 (ALDH2), crucial enzymes in the safe pathway.^36^ This optimizes alcohol metabolism, reducing the production of toxic acetaldehyde and reactive oxygen species.^47^ For lipid reduction, it downregulates the fatty acid uptake gene CD36 and upregulates fatty acid oxidation genes Ppar-α and Cpt-1, effectively reversing alcohol-induced hepatic steatosis.^47^ At the “antioxidant” level, it activates the core antioxidant defense hub of the body—the transcription factor Nrf2—thereby initiating its downstream pathways. This enhances the activity of multiple antioxidant enzymes such as superoxide dismutase­(SOD) and glutathione peroxidase, building a robust cellular defense system.^47^ Furthermore, *L. pentosus* LTJ12 and *Pediococcus acidilactici* (*P. acidilactici*) LTJ28, isolated from Chinese baijiu fermentation substrates, can reduce endotoxin-induced liver damage by 50% by regulating alcohol-metabolizing enzymes, alleviating oxidative stress, and restoring the function of the gut-liver axis.^61^ This study successfully screened strains with potent hepatoprotective functions from an extreme, high-concentration ethanol environment.^61^ It not only broadens the resource scope of PDFC but also suggests that these strains may have evolved unique metabolic and protective mechanisms during adaptation to fermentation stress.^60,61^ This tolerance, which has evolved through long-term coevolution with an alcohol-rich environment, makes these strains a unique resource for screening alcohol-tolerant probiotics, offering distinct advantages in the development of functional foods (such as alcoholic beverages containing active probiotics) designed to intervene in alcohol-induced liver injury.^60,61^ Their value lies in providing the theoretical and resource foundation for holistic intervention strategies based on the “microbiome-gut-liver” axis.^47,60,61^

Multiple studies indicate that PDFC demonstrate significant potential for regulating blood lipids and cholesterol, with mechanisms involving direct assimilation, enhanced excretion, and modulation of hepatic metabolism.^94^ For example, fermented oats rich in β-glucan specifically enrich *Lp. plantarum* strains that not only express bile salt hydrolase but also synergize with oat β-glucan to effectively promote intestinal cholesterol excretion and regulate hepatic cholesterol metabolism pathways.^94,131^ *Lp. plantarum* COORG 4 and *L. pentosus* COORG 7, isolated from fermented sorghum products, also demonstrated potent cholesterol assimilation capabilities in vitro models, with clearance rates of 74.12 and 68.26% for egg yolk cholesterol, respectively.^57^ This highly efficient assimilation is closely linked to the strain’s adaptive metabolism toward specific carbon sources in the sorghum substrate—components such as EPS synthesized during the utilization of sorghum polysaccharides may further enhance the efficiency of cholesterol binding and clearance.^57^ Mechanistic studies further reveal that *Lf. fermentum* FZU501, isolated from red yeast rice wine, reduces cholesterol levels in high-fat diet mice through multigenic synergistic regulation: it upregulates the Ldlr (low-density lipoprotein receptor) gene to enhance hepatic uptake of low-density lipoprotein cholesterol, upregulates the Cyp7a1 (cholesterol 7α-hydroxylase) gene to promote cholesterol conversion to bile acids and excretion, and mildly inhibits Hmgcr (HMG-CoA reductase) gene expression, regulating cholesterol synthesis at its source.^47^ This mode of action complements classic statins: PDFC not only mildly inhibit cholesterol synthesis but simultaneously enhance its reverse transport and biotransformation pathways, achieving “multi-pathway programming” of lipid metabolism.^47^ However, the practical translation potential of these findings warrants caution. Most existing studies are in vitro experiments or animal models, and the dose–response relationship, bioavailability, and long-term efficacy in humans still require confirmation through large-scale clinical trials.

In dietary management of hypertension, the unique role of PDFC extends beyond regulating the gut microbiota and reducing inflammation;^132,133^ it is further demonstrated by its synergistic effects with the cereal matrix. Probiotics in traditional fermented grain beverages secrete proteases during fermentation, hydrolyzing grain proteins to produce ACE-I peptides (such as Leu-Arg-Ala derived from rice).^57,134^ This “grow-and-release” mechanism enables the sustained production of antihypertensive peptides—often resulting in longer-lasting bioactivity compared to the direct intake of hydrolyzed products.^134^ Rice bran subjected to dual fermentation with fungi and LAB significantly reduced blood pressure in a rat model of stroke-prone spontaneous hypertension.^135^ This effect stems from the metabolic activity of the fermenting strains, which not only converts rice bran proteins into ACE-I peptides but also produces active metabolites that synergistically regulate adiponectin, leptin, and the adenosine monophosphate-activated protein kinase pathway, thereby achieving dual benefits of blood pressure reduction and metabolic improvement.^135^ In contrast, unfermented rice bran did not exhibit the same effects, indicating that the fermenting strains play a central role in transforming ordinary grains into functional foods, which is precisely what distinguishes PDFC from ordinary dietary components.^135^ Furthermore, acetic acid, which is abundant in grain vinegar produced by acetic acid bacteria fermenting grains, has also been shown to possess significant blood pressure-lowering activity.^136^

### 4.2 GI Diseases

Strains derived from various traditionally fermented grains each offer distinct advantages in terms of antimicrobial activity, antioxidant properties, and gut colonization, collectively enriching PDFC’s diverse resource library for gastrointestinal health interventions.^50,51,68^ In terms of microbiota regulation and infection prevention, *Lactobacillus* species derived from Indian fermented rice (such as *Lp. plantarum*, *Lacticaseibacillus casei*, and *Lf. fermentum*) significantly reduced *Clostridium difficile* colonization and infection rates in hospitalized elderly patients by modulating microbial community structure and enhancing the physical barrier.^50^ Additionally, *Meyerozyma* yeasts (such as *Meyerozyma guillermondii*) isolated from traditional fermented foods in the Indian state of Karnataka have also demonstrated strong antagonistic activity against pathogenic bacteria (>50%) and high gastrointestinal survival rates (>65%).^137^ Regarding antioxidant properties, LAB and yeasts from fermented grains (e.g., black glutinous rice and sorghum), along with their metabolites such as polyphenols, can enhance intestinal antioxidant enzyme activity and strengthen tight junctions, thereby alleviating intestinal mucositis.^68^ *Lp. plantarum* UBLP40, isolated from traditional Indian fermented foods, exhibits both antimicrobial and antioxidant properties.^138^ *Lacticaseibacillus paracasei* (*Lc. paracasei*) LUL:01, isolated from Lugri in the northwestern Himalayas, exhibits stronger antioxidant activity (2,2’-azino-bis (3-ethylbenzothiazoline-6-sulfonic acid) scavenging rate of 57.74%) and intestinal colonization ability,^139^ and its safety has been verified through multiple indicators. This strain can survive stably in fermented milk for 28 days, providing a guarantee of stability for the application of PDFC in functional foods.^139^ With respect to anti-inflammatory mechanisms, *Lf. fermentum* MG7011, derived from fermented rice, significantly suppressed lipopolysaccharide-induced nitric oxide production in macrophages, with effects comparable to nitric oxide synthase inhibitors.^51^ Triptamines produced by LAB and fungi in fermented rice bran inhibit the expression of proinflammatory factors in macrophages via an aryl hydrocarbon receptor -dependent pathway.^140^ Notably, tryptamine production depends on the availability of tryptophan in the grain and the strain’s tryptophan metabolic enzyme system; this “substrate-metabolite” matching relationship demonstrates the potential of PDFC for the targeted production of functional metabolites.^140^ In addition, the *S. cerevisiae* Y-89, isolated from the traditional Indian fermented beverage “haria”, significantly improved colon length and reduced the disease activity index in a dextran sulfate sodium-induced colitis mouse model, while also exhibiting inhibitory effects against *Entamoeba histolytica*.^141^ The underlying mechanism of the aforementioned multitarget effects lies in the specific regulation of probiotic metabolism by cereal substrates.^139,140^ After cultivation of *Limosilactobacillus reuteri* (*Lr. reuteri*) WX-94 in wheat bran arabinan, its postbiotics were significantly more effective in alleviating intestinal inflammation and liver lesions than those produced by the same strain cultivated in glucose.^142,143^ This direct comparison demonstrates that the functional potential of the strain must be induced by a cereal substrate to be fully unleashed, highlighting the unique advantages of PDFC in terms of functional activation.^142,143^ Furthermore, this grain-strain interaction highlights the unique value of PDFC—they can transform the grain matrix into a “postbiotic” carrier rich in functional components such as organic acids and antioxidants.^142,143^ Taking fermented glutinous rice as an example, the bioactive compounds produced by PDFC can directly act on the intestinal epithelium, upregulating the expression of ZO-1 and occludin, and inhibiting inflammation and apoptosis, thereby enhancing intestinal barrier function.^144^ However, existing studies largely rely on acute or short-term intervention models and lack evidence-based support regarding the efficacy and safety of long-term interventions for chronic gastrointestinal diseases.^51,68^ Therefore, despite promising mechanistic research, the clinical translation of PDFC in gastrointestinal diseases requires rigorously designed human clinical trials to clarify dose–response relationships, individual response variability, and long-term benefits. Their functional stability and reproducibility must also be validated in real-world dietary settings. Future research should focus on establishing a complete causal chain linking “strain-fermentation process-active components-host phenotype” to advance this field from mechanism exploration toward precision nutrition applications.

### 4.3 CNS Diseases

PDFC and its metabolites exert neuroprotective effects by mediating communication via the gut-brain axis.^69,145,146^ LAB derived from fermented black barley and quinoa reshape the gut microbiota, thereby reducing lipopolysaccharide­(LPS) leakage and the toll-like receptor­(TLR)­4 inflammatory pathway it triggers; simultaneously, they strengthen the intestinal and blood-brain barriers and inhibit microglial activation, thereby alleviating hippocampal damage and improving cognitive function.^69,145^ *Lf. fermentum* and *L. pentosus*, derived from fermented chickpeas and barley, can release ferulic acid via the FAE pathway, produce GABA using glutamate as a substrate, and ferment cereal fiber to produce SCFAs. This multifunctional synergy gives them unique potential for modulating the gut-brain axis and improving neurological function.^146^ Furthermore, polyphenolic compounds such as 4-hydroxyphenylpyruvic acid, produced during the probiotic fermentation of rice, further enhance the anti-inflammatory and antioxidant effects.^6^ Current evidence suggests that PDFC have the potential to modulate neuroinflammation and cognitive function through multitarget regulation via the gut-brain axis;^69,145,146^ however, their preventive efficacy still requires further validation in healthy individuals and those in the predisease stage. Future research should focus on the systematic integration of the gut-brain axis network and individualized response patterns to advance the development of PDFC as a dietary intervention with well-defined neuroprotective functions.

### 4.4 Cancer

In the field of cancer prevention, PDFC shows great promise.^52,147^ Research indicates that *L. acidophilus* and *Bifidobacterium lactis* (*B. lactis*) derived from germinated brown rice can effectively inhibit the formation of colorectal precancerous lesions induced by 1,2-dimethylhydrazine/sodium dextran sulfate in rats.^52,147^ The mechanisms involve enhancing antioxidant enzyme activity, regulating the expression of apoptosis-related proteins, and reducing proinflammatory factor levels, thereby improving the carcinogenic microenvironment and inducing apoptosis in abnormal cells.^51,144^ *Bacillus* species derived from Indian pancake batter possess both antioxidant properties and high adhesion to HT29 colon cancer cells, demonstrating potential for intestinal targeting.^148^ Fermentation of rice bran by *Bifidobacterium longum* (*B. longum*) increases the anticancer metabolite N-δ-acetylornithine by 170-fold, while simultaneously reducing the procarcinogenic metabolite 4-cholestene-3-one and modulating the bile acid pathway.^149^ It is worth noting that fermentation does not always enhance the efficacy. Another in vitro animal study showed that unfermented rice bran reduced the rate of colonic epithelial erosion by 72%, whereas *B. longum*-fermented rice bran reduced it by only 40%—suggesting that the strain, grain, and disease state must be precisely matched to achieve the optimal protective effect.^150^

## 5 Applications of Cereal Probiotics and Their Metabolites in Animal Diseases

In the field of healthy livestock and poultry farming, PDFC is expected to replace antibiotics and play a significant role in maintaining the intestinal barrier, regulating the immune system, and promoting growth.^151,152^ Studies indicate that *Lp. plantarum* and *Lentilactobacillus buchneri* (*L. buchneri*) derived from fermented barley can work synergistically to alleviate intestinal damage caused by *Escherichia coli* K88 infection and suppress the expression of the proinflammatory cytokine IL-6, with *L. buchneri* exhibiting stronger immunomodulatory and antibacterial properties.^151^ Additionally, branched-chain SCFAs produced by *Lactobacillus* and *Megabacteria* fermentation of corn gluten-wheat bran substrates effectively stimulate piglet appetite, modulate the cytokine profile, and improve intestinal barrier function and nutrient absorption, thereby enhancing growth performance.^152^

In feed resource development, probiotics derived from fermented distillers’ grains promote the development of the spleen and lymph nodes in Guanling crossbred cattle and enhance growth performance and immune function by regulating choline metabolism, unsaturated fatty acid synthesis, and insulin resistance pathways.^153,154^ Simultaneously, these strains can also specifically regulate key gut microbiota, such as *Christensenellaceae* R-7 group and *mucor,* providing a basis for developing functional feeds.^155^ Furthermore, the nutritional value of distilled grains is significantly enhanced following synergistic fermentation by *Lp. plantarum* and **Bacillus subtilis*,* demonstrating the integrated advantages of probiotics in feed processing.^156^

However, the actual efficacy and potential risks of PDFC require objective evaluation.^157^ Certain strains may transition from a commensal to pathogenic states under specific conditions, posing host adaptation risks.^158^ More concerning is the potential for *Bacillus* species—a widely used probiotic group in agriculture—to serve as vectors for antibiotic resistance genes.^158^ Their potential spread within the environment and food chain constitutes a potential threat to public health.^159^ Therefore, while promoting the application of PDFC in animal husbandry, it is imperative to strengthen the verification of strain-specific functions, conduct long-term safety assessments, and monitor the risk of antibiotic resistance diffusion. This approach ensures their rational and sustainable use in livestock farming through scientific and prudent stance.

## 6 Outlook

The scope of PDFC applications is continuously expanding from traditional gut health to systemic health maintenance, demonstrating broad prospects.^32,160–162^ In terms of disease intervention, PDFC has shown potential for controlling tuberculosis infections—with *Staphylococcus hominis* MANF2 derived from Koozh, a traditional Indian fermented millet food, exhibiting a 97.7% inhibition rate against *Mycobacterium tuberculosis* ^160^—as well as skin health maintenance functions—*Monascus* derived from fermented red yeast rice exert multiple antiaging and reparative effects by promoting fibroblast proliferation, upregulating antiaging genes, and inhibiting melanin production.^161^ In the field of health and functional foods, *Lacticaseibacillus rhamnosus* (*Lr. rhamnosus*) Yoba 2012 simultaneously achieves high-density colonization (>10^8^ CFU/g) and aflatoxin removal (reduction by more than 1,000-fold) during fermentation, providing a viable pathway for enhancing the safety of cereal foods.^32^ Meanwhile, the gluten-hydrolyzing activity of *Bacillus tequilensis* AJG23, isolated from fermented grain dough, opens up new avenues for the development of functional foods for individuals with gluten sensitivity.^162,163^ It is worth noting that byproducts of grain processing, such as rice bran and wheat bran, have demonstrated significant efficacy in the treatment of various chronic diseases following fermentation with probiotics.^130,135,140,149^ This highlights the potential health benefits inherent in agricultural resources and provides a scientific basis for the functional upgrading of traditional agricultural products.

The application of PDFC still faces multiple challenges. Most current research remains in the preliminary stages, and its actual biological effects and clinical relevance in the context of complex diseases have yet to be validated.^61,78^ Maintaining high activity throughout the entire process—from fermentation and storage to delivery—is critical to the effectiveness of the intervention; however, factors such as industrial processing and the extreme pH levels, bile salts, temperature fluctuations, and nutrient depletion within the gastrointestinal tract can severely compromise the number and function of live bacteria.^159^ Given the limitations of live-culture preparations—such as poor stability, the need for cold-chain storage, inability to withstand heat processing, and safety concerns for immunocompromised individuals—postbiotics are emerging as a highly promising alternative due to their combined advantages in bioactivity, safety, and stability. Optimizing fermentation control and addressing industrial challenges, such as poor process reproducibility and microbial imbalance, will be key to advancing the practical application of PDFC.

Probiotic strains exhibit highly specific functions, making it difficult to directly extrapolate the significant effects of specific strains to other cereal fermentation sources or microbial combinations.^160^ Furthermore, their functions may be significantly influenced by the food matrix, fermentation processes, and the host’s individual microbial background.^133^ To advance the transition of PDFC from mechanistic research to clinical application, it will be necessary in the future to establish a standardized research framework that integrates “strain-substrate-process-function”: (1) identify PDFC with specific functions and elucidate the molecular structures of their bioactive metabolites; (2) identify key bioactive components through systematic screening and functional analysis; and (3) develop a precision-matching model linking strains, grains, and disease states. Building upon this foundation, tiered, multicenter human clinical trials should be conducted to establish personalized response prediction models using multiomics biomarkers. Concurrently, by integrating multidisciplinary approaches such as organoids, systems biology, and artificial intelligence, we aim to elucidate the “host-microbiome-metabolite” interaction network. Furthermore, collaborative efforts among academia, industry, and research institutions should be fostered to develop evidence-based functional evaluation and production standards, ultimately enabling the reliable application of PDFC in precision nutrition and health interventions.

To ensure the stability of the final product’s probiotic efficacy, a systematic approach is essential to elucidate how the intrinsic properties of the matrix and processing parameters synergistically regulate the growth and survival of probiotic bacteria. Strain screening and domestication are the cornerstones of ensuring stability.^164,165^ Indigenous strains isolated from traditional fermented grain foods typically exhibit greater processing tolerance due to their long-term adaptation to the grain environment.^164^ By subjecting strains to directed domestication under simulated processing stress conditions, mutant strains with enhanced stability can be obtained.^165^ In recent years, microencapsulation has been a key strategy for improving the processing and storage stability of probiotics. Incorporating probiotics into a cereal matrix or developing encapsulation systems can significantly enhance their survival rate in the gastrointestinal environment.^166^ Research on the use of agricultural and food byproducts as eco-friendly encapsulation materials has attracted significant attention: cereal alcohol-soluble proteins, owing to their unique self-assembly behavior, can be used to construct nanocarriers via methods such as desolvation precipitation and electrospinning for the encapsulation of probiotics and functional packaging.^167^ An extract enriched with β-glucan from *S. cerevisiae* cell walls was used for probiotic encapsulation; it demonstrated superior cell viability compared to sodium alginate and improved the processing and storage stability of the strains.^168^ Additionally, integrating strategies such as prebiotics, genetic engineering, and high-density inoculation can further mitigate damage to the strains caused by the processing,^169^ thereby providing diverse technical pathways for the development of high-performance probiotic foods.

Although PDFC offers unique advantages in terms of functional activation, live-culture formulations still face challenges, such as poor storage stability, the need for cold-chain transportation, and safety concerns. Against this backdrop, recent research has provided systematic evidence supporting the potential advantages of postbiotics derived from fermented grains over live probiotics. First, postbiotics derived from *Lp. plantarum* and *L. pentosus* in sourdough can withstand temperatures as high as 120 °C and remain stable during storage at room temperature, thereby overcoming the limitations associated with live bacteria.^170^ Second, postbiotics derived from oat-based fermentation significantly outperform conventional heat-inactivated probiotics at equivalent doses in alleviating liver damage, regulating lipid metabolism, and reducing inflammation.^171^ This demonstrates that their advantages stem not only from the safety and stability of the “inactivated” form but also from unique bioactive compounds generated through grain-strain interactions.^171^ Building on this foundation, strain specificity further enables the customization of postbiotic functions.^170^ Different strains fermenting the same grain substrate exhibit distinct, separable, and targetable functional differences: *Lp. plantarum* LP25 significantly inhibits body weight gain and the expression of lipid synthesis genes (FASN and SREBF1), whereas *Pediococcus pentosaceus* (*P. pentosaceus*) PP18 is more effective at repairing the intestinal barrier; such precise targeting is difficult to achieve with live bacteria.^170^ Furthermore, postbiotics are not constrained by the survival conditions of live bacteria and can be directly blended with functional components in grains to improve texture and enhance antioxidant properties.^172^ Consequently, due to their direct mechanism of action, customizable targets, and strong compatibility with grains, postbiotics have opened up a new dimension in grain-based functional foods. However, while postbiotics themselves are generally safe, contamination may lead to a range of health issues.^173^ Although postbiotics derived from a single microbial source exhibit some efficacy, combining postbiotics from different sources or supplementing them in conjunction with probiotics may be more effective than supplementation with a single source, particularly in multitarget health applications.^172^

Synthetic biology, multiomics, and artificial intelligence are revolutionizing traditional fermentation paradigms, with efficient screening and rational design significantly improving the efficiency of constructing high-performance strains.^30^ However, the metabolic restructuring caused by genetic modification leads to fluctuations in the fermentation performance, introducing new challenges for process control. Intelligent methods such as data visualization and digital twins enhance optimization efficiency. This presents new opportunities for PDFC research: on the one hand, these technologies can be leveraged to identify functional strains and bioactive metabolites in traditional fermented grains and elucidate their interaction mechanisms with the grain matrix; on the other hand, grain-specific fermentation process models must be established to address metabolic fluctuations following strain modification, thereby driving the transition of PDFC from empirical screening to rational design.

## Data Availability

All data of this article are included within the article. It can also be requested from the corresponding author or first author.

## Abbreviation:

ACC: acetyl-CoA carboxylase
ACE: angiotensin-converting enzyme
ACE-I: angiotensin-converting enzyme inhibitory
ACO: acyl-CoA oxidase
ADH2: alcohol dehydrogenase 2
ALDH2: acetaldehyde dehydrogenase 2
*A. oryzae*: *Aspergillus oryzae*
Arg1: arginase-1
*B. animalis subsp. Lactis*: *Bifidobacterium animalis subsp. Lactis*
*B. lactis*: *Bifidobacterium lactis*
*B. longum*: *Bifidobacterium longum*
*B.* sp.: *Bifidobacterium* sp.
BAR: bile acid receptor
Bax: Bcl-2-associated X protein
Bcl-2: B-cell lymphoma-2
CAT: catalase
caspase-3: cysteinyl aspartate specific proteinase-3
CNS: central nervous system
CPT1: carnitine palmitoyltransferase I
CYP2E1: cytochrome p450 family 2 subfamily E member 1
CYP7A1: cytochrome P450 family 7 subfamily A member 1
DPPH: 2,2-diphenyl-1-picrylhydrazyl
EPS: exopolysaccharides
FAE: feruloyl esterase
FAS: fatty acid synthase
GABA: gamma-aminobutyric acid
GI: gastrointestinal
GPR43: G-protein-coupled receptor 43
GPR109A: G protein-coupled receptor 109A
GSH: glutathione
iNOS: inducible nitric oxide synthase
IL-10: interleukin-10
IL-18: interleukin-18
IL-1β: interleukin-1β
IL-6: Interleukin-6
LAB: lactic acid bacteria
*L. acidophilus*: *Lactobacillus acidophilus*
*L. brevis*: *Levilactobacillus brevis*
*L. buchneri*: *Lentilactobacillus buchneri*
*L. pentosus*: *Lactiplantibacillus pentosus*
*L. mesenteroides*: *Leuconostoc mesenteroides*
*Lc. paracasei*: *Lacticaseibacillus paracasei*
LDLR: low-density lipoprotein receptor
*Lf. fermentum*: *Limosilactobacillus fermentum*
*Lp. plantarum*: *Lactiplantibacillus plantarum*
LPS: lipopolysaccharide
*Lr. reuteri*: *Limosilactobacillus reuteri*
*Lr. rhamnosus*: *Lacticaseibacillus rhamnosus*
MALDI-TOF MS: matrix-assisted laser desorption/ionization time-of-flight mass spectrometry
MCT-1: monocarboxylate transporter 1
MCT-2: monocarboxylate transporter 2
*P. acidilactici*: *Pediococcus acidilactici*
*P. pentosaceus*: *Pediococcus pentosaceus*
PDFC: probiotics derived from fermented cereals
PPAR-α: peroxisome proliferator-activated receptor alpha
PPAR-δ: peroxisome proliferator-activated receptor delta
PPAR-γ: peroxisome proliferator-activated receptor gamma
ROOH: organic hydroperoxide
*S. cerevisiae*: *Saccharomyces cerevisiae*
SCFA: short-chain fatty acids
SLC16A1: solute carrier family 16 member 1
SLC16A7: solute carrier family 16 member 7
SOD: superoxide dismutase
SREBP-1c: sterol regulatory element-binding protein 1c
TLR: toll-like receptor
TNF-α: tumor necrosis factor alpha
*W. cibaria*: *Weissella cibaria*
